# Ozoralizumab-Induced Multifocal Demyelinating Neuropathy in a Patient With Rheumatoid Arthritis

**DOI:** 10.7759/cureus.98835

**Published:** 2025-12-09

**Authors:** Yuya Ikegawa, Yuichi Hamada, Naoki Hayakawa, Kazuto Nishinaka

**Affiliations:** 1 Neurology, Sumitomo Hospital, Osaka, JPN

**Keywords:** demyelinating diseases, ozoralizumab, peripheral nervous system diseases, rheumatoid arthritis, tumor necrosis factor inhibitors

## Abstract

Tumor necrosis factor-α (TNF-α) inhibitors can induce demyelinating polyneuropathy, but this has not been reported with ozoralizumab. We report a case of a 57-year-old male with rheumatoid arthritis who developed paresthesia in the left hand, bilateral wrist drop, and reduced grip strength after starting ozoralizumab. Nerve conduction studies showed bilateral radial nerve conduction blocks consistent with demyelination. Symptoms persisted after discontinuation of ozoralizumab but resolved after two courses of intravenous immunoglobulin therapy, with complete clinical and electrophysiological recovery. This case suggests that ozoralizumab may trigger multifocal demyelinating neuropathy.

## Introduction

Tumor necrosis factor-α (TNF-α) inhibitors have greatly enhanced the management of inflammatory diseases such as rheumatoid arthritis (RA) and inflammatory bowel disease [1]. However, they have also been associated with demyelinating disorders affecting the central nervous system (CNS) or peripheral nervous system (PNS), including multiple sclerosis, optic neuritis, Guillain-Barré syndrome, chronic inflammatory demyelinating polyneuropathy, and multifocal motor neuropathy [2-4].

Ozoralizumab, a new TNF-α inhibitor, was approved for treating RA in Japan in 2022, but no PNS demyelinating disease associated with its use has been reported. This report presents the initial instance of multifocal demyelinating neuropathy possibly triggered by ozoralizumab in a patient with RA.

## Case presentation

A 57-year-old Japanese man presented with a 2-month history of left-hand paresthesia, bilateral wrist drop, and reduced grip strength. He had a 9-year history of RA, which remained in remission following treatment with methotrexate, tacrolimus, and tocilizumab. Ozoralizumab was initiated 7 months before presentation for a relapse of joint symptoms. Neurological symptoms appeared 5 months later, persisting for 2 months despite discontinuation of ozoralizumab.

Neurological examination showed bilateral weakness in muscles innervated by the median and radial nerves. Grip strength measured 9/7 kg (right/left). Muscle strength was graded 5/5 bilaterally in the deltoid, biceps brachii, and triceps brachii; 4+/5 on the right and 2+/5 on the left in the wrist flexor (WF); 2/5 on the right and 5/5 on the left in the wrist extensor (WE); 4/5 on the right and 2/5 on the left in the abductor pollicis brevis (APB); 1/5 bilaterally in the extensor digitorum (ED); 5/5 bilaterally in the first dorsal interosseous and abductor digiti minimi; 4-/5 on the right and 2+/5 on the left in the flexor digitorum profundus (FDP) of the first finger; 5/5 bilaterally in the FDP of the fourth finger; and 4/5 on the right and 2/5 on the left in the flexor pollicis longus (FPL) using the Medical Research Council (MRC) scales. No lower extremity weakness was present. Paresthesia affected the first to fourth fingers of the left hand. Cranial nerve function and gait were normal.

Blood tests indicated elevated levels of RA-related antibodies, including rheumatoid factor (RF) at 22 IU/mL (reference: <15 IU/mL), anti-cyclic citrullinated peptide antibody at 116 IU/mL (reference: <4.5 IU/mL), and antinuclear antibody at a titer of 1:80 (reference: <1:40). Other autoimmune-related antibodies, such as anti-double-stranded DNA antibody, anti-neutrophil cytoplasmic antibody, and anti-Sjögren's syndrome-related antigen, were all negative. Anti-ganglioside antibodies, such as IgG and IgM for GM1, GD1a, GD1b, GT1b, GQ1b, GA1, GM2, GM3, GD3 and GD2, were also negative. The serum levels of glucose, vitamin B1, and vitamin B12 were normal, but the level of folic acid was slightly low at 3.5 ng/mL (reference: >4.0 ng/mL). Cerebrospinal fluid analysis showed albuminocytologic dissociation, with a normal cell count of 2/µL (reference: <5/µL) and an elevated protein level of 60.5 mg/dL (reference: 8-43 mg/dL). The IgG index was slightly elevated at 0.7 (reference: <0.65).

The results of nerve conduction studies (NCS) are shown in Tables 1-2. The sensory nerves were normal. Bilateral median motor nerve conduction studies (MNCS) were within the normal range. However, the muscle weakness in the left hand suggested damage to the left median motor nerve. It was suspected that the left median nerve was damaged in the upper arm proximal to the studied segment. Bilateral radial MNCS showed conduction blocks (CB) within the upper arm fulfilling the European Academy of Neurology/Peripheral Nerve Society criteria [5]. We diagnosed these localized lesions of the motor nerves as the cause of the symptoms. Given that multiple nerves were affected and symptoms were progressing over time, we determined that the cause is not compression.

**Table 1:** Motor nerve conduction studies. Motor nerve conduction studies were performed on the median and radial nerves. Asterisks indicate values considered abnormal according to the European Academy of Neurology/Peripheral Nerve Society criteria [5]. After treatment, the compound muscle action potential amplitudes of the median nerves increased, and the radial motor nerve conduction blocks resolved. DL, distal latency; Amp, amplitude; CV, conduction velocity; NR, not recorded

Nerve	Time Course	Site	DL (ms)	Amp (mV)	CV (m/s)
Left Median	Before treatment	Wrist	3.3	6.8	-
		Elbow		6.5	55
	After treatment	Wrist	3.6	9.3	-
		Elbow		8.9	54
Right Median	Before treatment	Wrist	3.4	5.8	-
		Elbow		3.7	46
	After treatment	Wrist	3.2	8.4	-
		Elbow		6.0	49
Left Radial	Before treatment	Forearm	1.8	4.2	-
		Upper arm		1.5*	49*
		Axilla		1.4*	43*
	After treatment	Forearm	2.9	4.7	-
		Upper arm		3.0	42*
		Axilla		NR	-
Right Radial	Before treatment	Forearm	2.8	6.4	-
		Upper arm		5.2	58
		Axilla		1.8*	23*
	After treatment	Forearm	2.9	5.4	-
		Upper arm		4.1	54
		Axilla		2.5*	26*

**Table 2:** Sensory nerve conduction studies. Sensory nerve conduction studies were performed on the median and radial nerves. After treatment, sensory nerve action potential amplitudes showed slight improvement. Sensory nerve conduction studies were performed using the orthodromic method. DL, distal latency; Amp, amplitude; CV, conduction velocity

Nerve	Time course	DL (ms)	Amp (µV)	CV (m/s)
Left Median	Before treatment	2.5	28.4	59
	After treatment	2.3	30.5	65
Right Median	Before treatment	2.5	28.7	64
	After treatment	2.6	36.4	63
Left Radial	Before treatment	2.1	24.5	67
	After treatment	2.1	31.2	66
Right Radial	Before treatment	2.2	34.8	64
	After treatment	2.2	33.5	65

**Figure 1:**
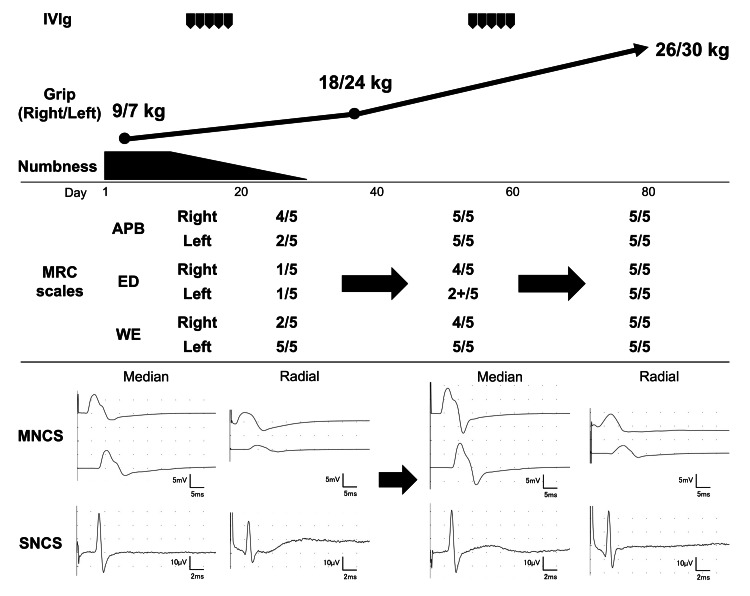
Clinical course and electrophysiological changes. Changes in grip strength, numbness, and muscle strength (APB, ED, and WE on the MRC scale) following intravenous immunoglobulin therapy are shown. Nerve conduction waveforms of the left median and radial nerves before and after treatment are also presented. IVIg: intravenous immunoglobulin; MNCS: motor nerve conduction study; MRC: Medical Research Council; SNCS: sensory nerve conduction study

Ozoralizumab was considered the likely cause based on previous reports. Neurological symptoms did not improve after discontinuation of ozoralizumab alone. Intravenous immunoglobulin (IVIg) therapy was initiated (400 mg/kg daily for 5 days) 12 days after the initial presentation. By day 34, grip strength improved to 18/24 kg (right/left) with full WF and APB recovery to 5/5 bilaterally on the MRC scale. However, muscle strength in the WE (4/5 on the right and 5/5 on the left), FDP of the first finger (4/5 bilaterally), ED (4/5 on the right and 2+/5 on the left), and FPL (5/5 on the right and 4/5 on the left) did not achieve complete recovery on the MRC scale. Paresthesia in the left hand was also partially resolved. Owing to the persistent neurological symptoms, a second course of IVIg was administered using the same regimen, 53 days after the initial presentation. By day 85, grip strength improved to 26/30 kg (right/left) and returned to baseline. Muscle strength in the WE, FDP of the first finger, ED, and FPL improved to 5/5 bilaterally on the MRC scale. Paresthesia also resolved. Follow-up NCS demonstrated a corresponding improvement (Tables 1-2; Figure 1). The patient has been symptom-free since, and RA remains in remission with upadacitinib therapy.

## Discussion

Demyelinating neuropathy has been documented in approximately 0.6% of patients receiving TNF-α inhibitors, with a median onset of 2-60 months. While many patients show improvement after the discontinuation of TNF-α inhibitors, some respond well to IVIg therapy [6]. In our case, symptoms persisted despite TNF-α inhibitor withdrawal but resolved after IVIg administration. The absence of previous reports involving ozoralizumab may reflect its limited clinical use, rapid and targeted delivery via albumin binding, and reduced immunogenicity due to the lack of an Fc region [7].

The patient exhibited multifocal neuropathy, with discrete weakness and paresthesia across multiple regions. Although rheumatoid vasculitis can cause multifocal neuropathy, it is axonal neuropathy [8]. However, the neuropathy in our case was demyelinating rather than axonal. The rapid and complete improvement of symptoms with treatment, along with improvement in CB on MNCS, is inconsistent with axonal damage. This suggests that the CB observed initially was not pseudo-CB as seen in axonal damage due to vasculitis or ischemia, but rather true CB caused by demyelination. Although sensory nerve action potential amplitudes remained within the normal range both before and after treatment, they demonstrated an overall upward trend, suggesting the possibility of initial impairment. CB can result from compression, but this was unlikely in our case because multiple nerves were affected and abnormalities persisted. These findings support ozoralizumab-induced demyelination neuropathy rather than rheumatoid vasculitis. This case highlights the importance of NCS in patients with RA with associated neuropathy, as they help distinguish demyelination from axonal types. Clinicians should suspect TNF-α inhibitor-induced neuropathy when demyelination is detected.

TNF-α contributes to demyelination through various mechanisms, including cytotoxic effects on the vascular endothelium, enhancement of macrophage phagocytosis, and direct cytotoxic damage to Schwann cells and myelin sheaths [2]. Paradoxically, TNF-α inhibitors, designed to suppress TNF-α signaling, can induce demyelinating neuropathy. This paradoxical effect may result from the inhibitors increasing autoreactive T-cell activity by reducing T-cell apoptosis and enhancing antigen-presenting cell function, consequently promoting TNF-α signaling within the CNS [9]. Furthermore, these inhibitors are unable to penetrate the blood-brain barrier or suppress increased TNF-α signaling within the CNS, potentially leading to exacerbation of CNS demyelination [10]. While limited, the evidence suggests a similar mechanism could occur in the PNS. In other words, TNF-α inhibitors may struggle to cross the blood-nerve barrier, potentially intensifying TNF-α activity in the PNS and exacerbating inflammatory demyelination.

## Conclusions

Although demyelinating neuropathy is a recognized complication of other TNF-α inhibitors, it has not been reported with ozoralizumab. Our case findings suggest that ozoralizumab may also trigger demyelinating neuropathy. RA, an indication for TNF-α inhibitor use, can cause vasculitic neuropathy. Given the increased risk of neuropathy in patients with RA treated with TNF-α inhibitors, routine neurological assessment is recommended. Determining the cause of neuropathy in such patients is crucial for appropriate management. NCS is useful in differentiating between vasculitic neuropathy and TNF-α inhibitor-induced demyelinating neuropathy. When TNF-α inhibitor-induced neuropathy is suspected, clinicians should consider discontinuing the drug and administering IVIg for persistent symptoms.
